# Targeted disruption of *Tbc1d20* with zinc-finger nucleases causes cataracts and testicular abnormalities in mice

**DOI:** 10.1186/s12863-014-0135-2

**Published:** 2014-12-05

**Authors:** Anna Kyunglim Park, Ryan P Liegel, Adam Ronchetti, Allison D Ebert, Aron Geurts, Duska J Sidjanin

**Affiliations:** Department of Cell Biology, Neurobiology and Anatomy, Medical College of Wisconsin, 8701 Watertown Plank, Milwaukee, WI 53226 USA; Department of Physiology, Medical College of Wisconsin, 8701 Watertown Plank, Milwaukee, WI 53226 USA; Human and Molecular Genetics Center, Medical College of Wisconsin, 8701 Watertown Plank, Milwaukee, WI 53226 USA

**Keywords:** *TBC1D20*, Loss-of-function, Zinc-finger nuclease, Blind-sterile, Spermatogenesis, Warburg Micro Syndrome

## Abstract

**Background:**

Loss-of-function mutations in *TBC1D20* cause Warburg Micro syndrome 4 (WARBM4), which is an autosomal recessive syndromic disorder characterized by eye, brain, and genital abnormalities. *Blind sterile* (*bs*) mice carry a *Tbc1d20-null* mutation and exhibit cataracts and testicular phenotypes similar to those observed in WARBM4 patients. In addition to *TBC1D20*, mutations in *RAB3GAP1, RAB3GAP2* and *RAB18* cause WARBM1-3 respectively. However, regardless of which gene harbors the causative mutation, all individuals affected with WARBM exhibit indistinguishable clinical presentations. In contrast, *bs, Rab3gap1*^*-/-*^*,* and *Rab18*^*-/-*^ mice exhibit distinct phenotypes; this phenotypic variability of WARBM mice was previously attributed to potential compensatory mechanisms. *Rab3gap1*^*-/-*^ and *Rab18*^*-/-*^ mice were genetically engineered using standard approaches, whereas the *Tbc1d20* mutation in the *bs* mice arose spontaneously. There is the possibility that another unidentified mutation within the *bs* linkage disequilibrium may be contributing to the *bs* phenotypes and thus contributing to the phenotypic variability in WARBM mice. The goal of this study was to establish the phenotypic consequences in mice caused by the disruption of the *Tbc1d20* gene.

**Results:**

The zinc finger nuclease (ZFN) mediated genomic editing generated a *Tbc1d20* c.[418_426del] deletion encoding a putative TBC1D20-ZFN protein with an in-frame p.[H140_Y143del] deletion within the highly conserved TBC domain. The evaluation of *Tbc1d20*^*ZFN/ZFN*^ eyes identified severe cataracts and thickened pupillary sphincter muscle. *Tbc1d20*^*ZFN/ZFN*^ males are infertile and the analysis of the seminiferous tubules identified disrupted acrosomal development. The compound heterozygote *Tbc1d20*^*ZFN/bs*^ mice, generated from an allelic *bs/+* X *Tbc1d20*^*ZFN/+*^ cross, exhibited cataracts and aberrant acrosomal development indicating a failure to complement.

**Conclusions:**

Our findings show that the disruption of *Tbc1d20* in mice results in cataracts and aberrant acrosomal formation, thus establishing *bs* and *Tbc1d20*^*ZFN/ZFN*^ as allelic variants. Although the WARBM molecular disease etiology remains unclear, both the *bs* and *Tbc1d20*^*ZFN/ZFN*^ mice are excellent model organisms for future studies to establish TBC1D20-mediated molecular and cellular functions.

## Background

Warburg Micro syndrome (WARBM) is a genetically heterogeneous autosomal recessive syndromic disorder characterized by eye, brain, and genital abnormalities [1]. Mutations in *RAB3GAP1*, *RAB3GAP2*, *RAB18*, and *TBC1D20* genes cause WARBM1, WARBM2, WARBM3, and WARBM4 forms respectively [2-5]. Regardless which of the four genes harbors the causative mutation, all WARBM individuals present with indistinguishable clinical features [1,5]. Eye abnormalities in WARBM children are characterized by congenital cataracts, microphakia, microcornea, microphthalmia, optic nerve atrophy, and small, atonic pupils [6,7]. Postnatal microcephaly, predominantly frontal polymicrogyria, corpus callosum hypogenesis, enlarged subdural spaces, cerebellar vermis hypoplasia are brain characteristics in the affected WARBM children; these abnormalities are accompanied by seizures and severe intellectual disability [8-10]. Microgentialia is present in both the WARBM affected boys and girls [1,7,9]. In addition to eye, brain and genital abnormalities, WARBM children also exhibit hypotonia of truncal muscles, as well as spasticity of the limbs resulting in the inability to walk, sit, or crawl, and ultimately resulting in quadriplegia [1].

Mouse models of human genetic disorders are excellent resources for elucidation of the molecular and cellular disease etiologies. Recently, we reported that *blind sterile* (*bs*) mice, initially identified over 30 years ago as a spontaneous autosomal recessive mouse mutation exhibiting cataracts [11,12] and male infertility [13,14], carry a loss of function mutation in the *Tbc1d20* gene [5]. The *bs* mice recapitulate the lens and testicular phenotypes observed in the WARBM4 children, although no morphological brain abnormalities were noted [5]. *Rab3gap1*^*-/-*^ mice do not exhibit any morphological abnormalities of the eyes, brain, or genitalia, but exhibit synaptic exocytosis abnormalities [15]. Recently, it was shown that *Rab18*^*-/-*^ mice exhibit cataracts, atonic pupils, and progressive hind limb weakness associated with accumulations of neurofilament and microtubules in the synaptic terminals [16]. This phenotypic variability between mice with disrupted WARBM genes has been previously attributed to gene-specific and species-specific compensatory mechanisms present in mice [4,5].

*Rab3gap1*^*-/-*^ and *Rab18*^*-/-*^ mice are mouse models that were genetically engineered using standard approaches [15,16]. In contrast, the *Tbc1d20* mutation in the *bs* mouse arose spontaneously [11]. Our genetic analysis of the *bs* mice identified a 416 kb genomic region in linkage disequilibrium within the *bs* locus [5]. The analysis of the *bs* critical region identified 16 RefSeq candidate genes and further evaluation of the candidate genes focused on the sequencing of the exons and exon/intron boundaries as well as RT-PCR analysis and subsequent sequencing of the open reading frames [5]. This approach identified a c.[691 T > A; 692_703del] mutation in the *Tbc1d20* gene as causing the *bs* phenotype; subsequent functional analysis of the TBC1D20-bs protein determined that the *bs* mutation results in the loss of TBC1D20 functional [5]. Given that we did not sequence the entire 416 kb *bs* critical region, we cannot eliminate the possibility that another mutation not residing within the exon/intron regions or open reading frames of the 16 candidate genes, but resides within the *bs* linkage disequilibrium region, may be contributing to the phenotypic differences between the *bs, Rab3gap1*^*-/-*^, and *Rab18*^*-/-*^ mice.

As a part of this study, we set out to unequivocally establish the phenotypic consequences caused by the disruption of the *Tbc1d20* gene. We utilized the zinc-finger nuclease (ZFN)-mediated genomic editing approach to generate the *Tbc1d20*^*ZFN/ZFN*^ mice. Our results show that the *Tbc1d20*^*ZFN/ZFN*^ mice exhibit cataracts and testicular phenotypes indistinguishable from the cataract and testicular phenotypes identified in the *bs* mice. Additionally, the complementation analysis confirmed that the *bs* and *Tbc1d20*^*ZFN/ZFN*^ mice are allelic variants.

## Results and discussion

### ZFN-mediated disruption of the *Tbc1d20* locus

**Figure 1:**
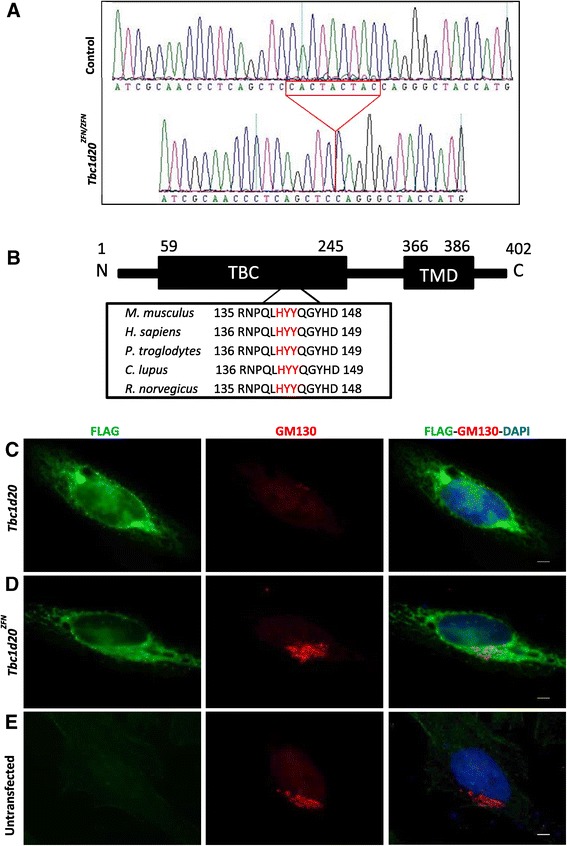
**The evaluation of the** ***Tbc1d20*** ^***ZFN***^ **allele.** ZFN-mediated genomic editing resulted in the *Tbc1d20* ^*ZFN*^ transcript characterized by a 9 bp c.[418_426del] deletion **(A)**. The *Tbc1d20* ^*ZFN*^ allele encodes the TBC1D20-ZFN mutant protein with an in-frame 3 amino acid p.[H140_Y143del] deletion within a highly evolutionarily conserved TBC domain. Missing amino acids are depicted in red **(B)**. **(C)** Overexpression of FLAG-tagged TBC1D20-WT (green) led to a disruption of the Golgi as evident by the punctate GM130 immunostaining (red). **(D)** Overexpression of the FLAG-tagged TBC1D20-ZFN protein (green) did not disrupt GM130 immunostaining of the Golgi and did not differ from GM130 immunostaining of the untransfected HeLa cell **(E)**. DNA was stained with DAPI (blue). Scale bars = 5 μm.

The ZFN mediated targeting of the *Tbc1d20* gene (NM_024196) was designed to cut a 6 bp region within exon 4 (see Methods). This approach generated 3 *Tbc1d20*^*ZFN*^ founder mice with a 9 bp c.[418_426del] deletion (Figure 1A). The *Tbc1d20*^*ZFN*^ transcript encodes a putative TBC1D20-ZFN protein with an in-frame 3 amino acid deletion p.[H140_Y143del] within a highly evolutionarily conserved TBC domain (Figure 1B). TBC1D20 is an ER associated protein that functions as a GTPase activating protein (GAP) enhancing the GTP hydrolysis rate when bound to RAB1 or RAB2 [5,17,18]. It was shown previously that overexpression of mouse or human TBC1D20-WT protein results in the disruption of Golgi structures [5,17]. It was also shown that overexpression of the catalytically inactive mouse or human TBC1D20 proteins did not have an effect on the Golgi morphology [5,17]. Therefore, we proceeded to evaluate the effects of overexpression of the FLAG-tagged TBC1D20-WT and TBC1D20-ZFN proteins of Golgi structures in the HeLa cells. FLAG immunostaining confirmed the ER pattern of expression for both TBC1D20-WT and TBC1D20-ZFN proteins (Figure 1C-D). HeLa cells overexpressing of the FLAG-tagged TBC1D20-WT protein exhibited disrupted Golgi structures and only residual GM130 immunostaining (Figure 1C). In contrast, both untransfected (Figure 1E) and HeLa cells overexpressing the FLAG-tagged TBC1D20-ZFN protein exhibited similar GM130 immunostaining pattern (Figure 1D) suggesting that TBC1D20-ZFN did not disrupt Golgi structures. Therefore, these findings suggested that TBC1D20-ZFN catalytic function was disrupted.

### Eye, testicular, and brain phenotypes in *Tbc1d20*^*ZFN/ZFN*^ mice

**Figure 2:**
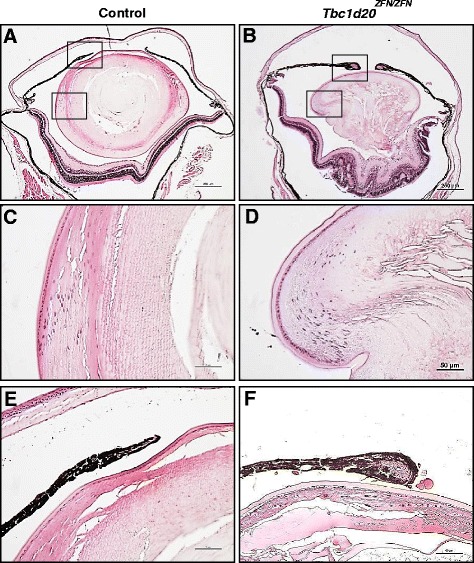
**The eye phenotypes in** ***Tbc1d20*** ^***ZFN/ZFN***^ **mice.** H&E analysis revealed severely disrupted P28*Tbc1d20* ^*ZFN/ZFN*^ eyes **(B)** eyes when compared to controls **(A)**; scale bars = 250 μm. *Tbc1d20* ^*ZFN/ZFN*^ vacuolated lenses exhibiting severely shortened and disorganized lens fiber cells **(D)** in contrast to highly organized lens fibers in control lenses **(C)**; scale bars = 50 μm. The *Tbc1d20* ^*ZFN/ZFN*^ mice exhibited thickened pupillary sphincter muscle **(F)** when compared to the pupillary sphincter muscled noted in control eyes **(E)**; scale bars = 50 μm.

The *Tbc1d20*^*ZFN/+*^ heterozygote mice did not phenotypically differ from the WT mice. The het to het breedings of the *Tbc1d20*^*ZFN/+*^ mice recovered *Tbc1d20*^*+/+*^ (n = 13), *Tbc1d20*^*ZFN/+*^ (n = 27), and *Tbc1d20*^*ZFN/ZFN*^ (n = 10) progeny and these ratios did not significantly differ, following a chi-squared test, from expected ratios for a Mendelian autosomal recessive locus. Following the eyelid opening around postnatal day P14, clinical eye evaluation identified nuclear cataracts only in *Tbc1d20*^*ZFN/ZFN*^ that by P28 progressed to total cataracts characterized by vacuoles present throughout the entire lens (not shown). Histological analysis of *Tbc1d20*^*ZFN/ZFN*^ eyes confirmed severely disrupted vacuolated lenses with ruptured lens capsule and lenticular material in the vitreal cavity (Figure 2B) although some lenticular material was also present in the anterior chamber (Figure 2F). Lens epithelial cells did not appear to exhibit any gross morphological abnormalities whereas cortical and nuclear fiber cells were severely shortened and disorganized (Figure 2D). Although retinal dismorphology and rosetting were evident in *Tbc1d20*^*ZFN/ZFN*^ eyes (Figure 2B), the retina was laminated suggesting that rosetting may have been caused by the lens rupture and not by a defect in retinal development. *Tbc1d20*^*ZFN/ZFN*^ eyes also exhibited thickened pupillary sphincter muscle (Figure 2F) that was not previously identified in *bs* eyes [5] suggesting that this TBC1D20-associated phenotype may be influenced by genetic modifiers.

**Figure 3:**
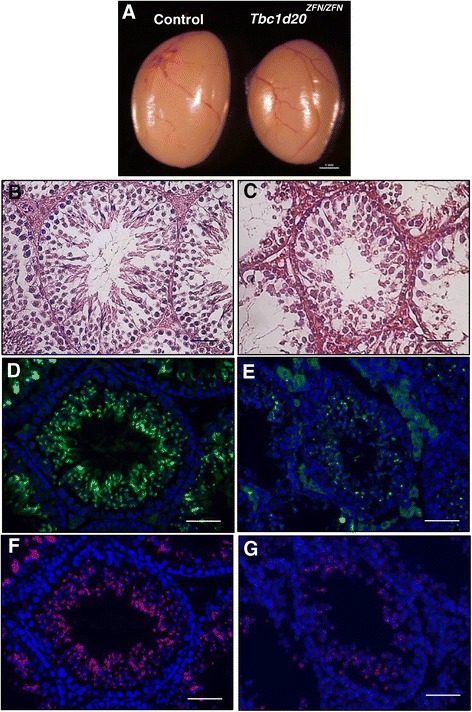
**The testicular phenotypes in** ***Tbc1d20*** ^***ZFN/ZFN***^ **mice.** *Tbc1d20* ^*ZFN/ZFN*^ testes appeared smaller in size when compared to controls **(A)**; scale bar = 1 mm. H&E analysis identified disorganized *Tbc1d20* ^*ZFN/ZFN*^ seminiferous tubules **(C)** when compared to highly organized seminiferous tubules in controls **(B)**; scale bars = 50 μm. TRA54 immunostaining (green) in control tubules revealed small punctae and crescent-shaped staining consistent with spermatocytes and round spermatids respectively **(D)** and in *Tbc1d20* ^*ZFN/ZFN*^ only TRA54 positive punctate staining was evident **(E)**. PNA staining of control tubules identified the presence of acrosomes **(F)**, whereas in *Tbc1d20* ^*ZFN/ZFN*^ only PNA positive punctate staining was noted **(G)**; scale bars = 25 μm. DNA was stained with DAPI (blue).

*Tbc1d20*^*ZFN/ZFN*^ females were able to produce litters and *Tbc1d20*^*ZFN/ZFN*^ males did not suggesting that the *Tbc1d20*^*ZFN/ZFN*^ males may be infertile. We proceeded to evaluate the *Tbc1d20*^*ZFN/ZFN*^ testes. Upon observation, the *Tbc1d20*^*ZFN/ZFN*^ testes appeared smaller in size when compared to control testes (Figure 3A). Histological evaluation revealed disorganized *Tbc1d20*^*ZFN/ZFN*^ seminiferous tubules (Figure 3C). Male infertility in TBC1D20-deficient *bs* mice was caused by a disruption in acrosomal formation [5,13,14], thus, we proceeded to evaluate the maturation of the spermatozoa in the *Tbc1d20*^*ZFN/ZFN*^ seminiferous tubules. Immunostaining with TRA54, a haploid sperm cell-specific antigen [19], of control seminiferous tubules revealed punctate (not shown) and crescent-shaped staining (Figure 3D) characteristic of spermatocytes and round spermatids respectively [19]. In contrast, immunostaining for TRA54 in *Tbc1d20*^*ZFN/ZFN*^ seminiferous tubules revealed only punctate staining (Figure 3E). Peanut agglutinin (PNA) is a marker for acrosomes [20]; PNA staining of the seminiferous tubules in the controls revealed a characteristic crescent acrosomal shape (Figure 3F) whereas in*Tbc1d20*^*ZFN/ZFN*^ seminiferous tubules only the PNA positive punctae were evident (Figure 3G). The observed testicular phenotypes of *Tbc1d20*^*ZFN/ZFN*^ were indistinguishable from the testicular phenotypes reported for the *bs* mice [5,13,14].

Evaluation of the *Tbc1d20*^*ZFN/ZFN*^ brains did not identify any gross morphological abnormalities (not shown). Collectively these findings indicated that in *Tbc1d20*^*ZFN/ZFN*^ mice eye and testicular phenotypes are fully penetrant without any brain morphological abnormalities consistent with findings previously reported for *bs* mice [5].

### Cellular phenotypes of *Tbc1d20*^*ZFN/ZFN*^ MEFs

**Figure 4:**
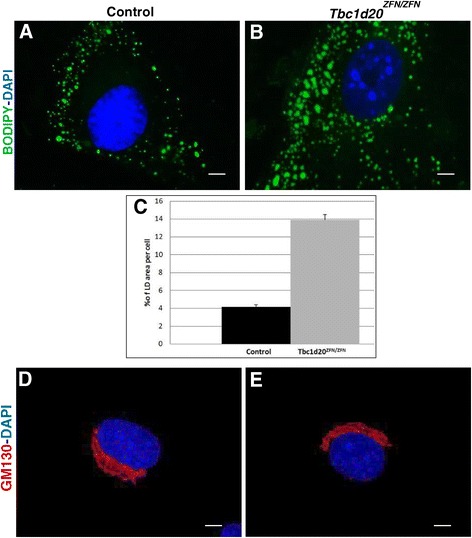
***Tbc1d20*** ^***ZFN/ZFN***^ **mEF cellular phenotypes.** Oleic acid treatment for 24 hr following staining with the neutral lipid dye BODIPY 493/503 revealed expanded LD structures in *Tbc1d20* ^*ZFN/ZFN*^ MEFs **(B)** when compared to control MEFs **(A)**. Quantification analyses shown in **(C)** identified that % of LD area per cell in *Tbc1d20* ^*ZFN/ZFN*^ (13.89 ± 1.23) was significantly greater (*P* < 0.001) than in control (4.16 ± 0.25) MEFs. *P* values were determined by Student’s *t* test and error bars represent SEM. GM130 immunostaining (red) revealed no Golgi differences between *Tbc1d20* ^*ZFN/ZFN*^ **(E)** and control MEFs **(D)**. DNA was stained with DAPI (blue). Scale bars = 5 μm.

An accumulation of enlarged lipid droplets (LDs) following oleic acid supplementation was the only cellular abnormality in the skin-derived TBC1D20-deficient fibroblasts from a WARBM4 patient [5]. Primary *bs* MEFs also exhibit an accumulation of enlarged LDs following treatment with oleic acid, but additionally the *bs* MEFs also exhibited enlarged Golgi structures [5]. Therefore, we proceeded to evaluate the LD and Golgi morphology in control and *Tbc1d20*^*ZFN/ZFN*^ MEFs. Our analysis confirmed a significant accumulation of enlarged LDs in the *Tbc1d20*^*ZFN/ZFN*^ MEFs (Figure 4B) when compared to the LDs in the MEFs from the control mice (Figure 4C) 24 h following oleic acid treatment and subsequent staining with the neutral lipid dye BODIPY 493/503. However, we did not observe any difference in the Golgi structures between control and *Tbc1d20*^*ZFN*^ MEFs following immunostaining with GM130 (Figure 4D and F). Western blot analysis confirmed there was no difference in levels of GM130 protein in control and *Tbc1d20*^*ZFN*^ MEF cell lysates (not show). Although *bs* MEFs exhibited enlargement of Golgi structures, Golgi structures in the TBC1D20-deficient skin fibroblasts from a WARBM4 patient did not differ from Golgi structures in control skin fibroblasts [5]. However, thickened Golgi ribbons were observed in HeLa cells following shRNA mediated *TBC1D20* knock-down [17]. Collectively these findings indicate that a spectrum of Golgi phenotypes is associated with TBC1D20 functional deficiency indicating that this phenotype is most likely influenced by genetic modifiers.

### Complementation analysis

**Figure 5:**
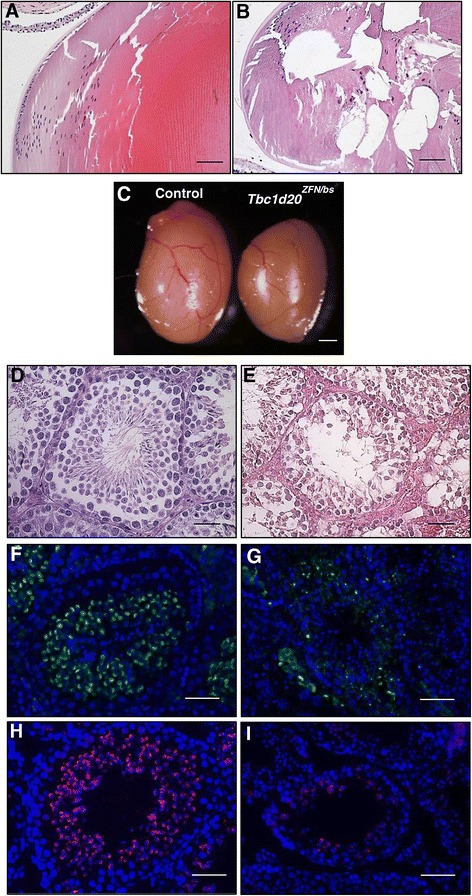
**Eye and testicular phenotypes in compound heterozygote** ***Tbc1d20*** ^***ZFN/bs***^ **mice.** H&E analysis revealed cataracts in *Tbc1d20* ^*ZFN/ZFN*^ lenses characterized by the presence of vacuoles **(B)** when compared to highly organized control lenses **(A)**; scale bars = 50 μm. *Tbc1d20* ^*ZFN/bs*^ testes appeared smaller in size when compared to controls **(C)**; scale bar = 1 mm. H&E analysis identified disorganized *Tbc1d20* ^*ZFN/bs*^ seminiferous tubules **(E)** when compared to highly organized seminiferous tubules in controls **(D)**; scale bars = 50 μm. Immunostaining with TRA54 (green) in control tubules revealed small punctae and crescent-shaped staining consistent with spermatocytes and round spermatids respectively **(F)** and in *Tbc1d20* ^*ZFN/bs*^ only TRA54 positive punctate staining was evident **(G)**. PNA positive acrosomes were evident in control tubules **(H)**, whereas in *Tbc1d20* ^*ZFN/ZFN*^ only PNA positive punctate staining was noted **(I)**; scale bars = 25 μm. DNA was stained with DAPI (blue).

To determine if *bs* and *Tbc1d20*^*ZFN*^ mice are allelic variants, we set up complementation breedings. A cross between *bs/+* and *Tbc1d20*^*ZFN/+*^ mice led to *Tbc1d20*^*ZFN/bs*^ (n = 4), *Tbc1d20*^*+/+*^ (n = 3), *Tbc1d20*^*ZFN/+*^ (n = 2), and *Tbc1d20*^*bs/+*^ (n = 3) progeny. Clinical eye evaluation (not shown) as well as histological eye analysis identified vacuolated cataracts in the *Tbc1d20*^*ZFN/bs*^ compound heterozygous mice (Figure 5B) phenotypically similar to the *Tbc1d20*^*ZFN/ZFN*^ cataracts (Figure 2B) as well as *bs* cataracts [5]. The compound heterozygous *Tbc1d20*^*ZFN/bs*^ mice did not exhibit pupillary thickening observed in *Tbc1d20*^*ZFN/ZFN*^ (not shown). The testes from the *Tbc1d20*^*bs/ZFN*^ compound heterozygote males appeared smaller in size when compared to controls (Figure 5C). Histological analysis revealed disorganized *Tbc1d20*^*ZFN/bs*^ seminiferous tubules (Figure 5). *Tbc1d20*^*ZFN/bs*^ seminiferous tubules immnunostaining with TRA54 (Figure 5G) and staining with PNA (Figure 5I) identified disrupted acrosomal formation phenotypically indistinguishable from the findings in *Tbc1d20*^*ZFN/ZFN*^ (Figure 3A,C,E and G) and *bs* males [5].

## Conclusions

In mice, the disruption of *Tbc1d20* results in vacuolated cataracts and a defect in acrosomal formation resulting in male infertility. At the cellular level, disruption of *Tbc1d20* resulted in an accumulation of LDs. Thickening of the pupillary sphincter muscle eye phenotypes and aberrant Golgi cellular phenotypes were not penetrant on all genetic backgrounds suggesting that these phenotypes, caused by disruption of *Tbc1d20,* may be influenced by genetic modifiers. Although molecular and cellular disease etiology caused by TBC1D20 functional deficiency in mice and humans remains unclear, *bs* and *Tbc1d20*^*ZFN/ZFN*^ mice are allelic variants and as such are excellent model organisms for future studies focusing on elucidating TBC1D20 function.

## Methods

### Mice

To target the mouse *Tbc1d20* (NM_024196.3) gene, ZFN plasmid design, assembly, validation and mRNA was done by the CompoZr Custom ZFN Service (Sigma). The ZFNs were designed to cut the c.[419ACTACT424] sequence within exon 4. The *Tbc1d20* targeting ZFN mRNA was injected into the B6D2F1/Crl (F1 het from C57BL/6 N and DBA2 strains) embryos, which were implanted into pseudo-pregnant females. Pups were genotyped using standard conditions with ZFN-F 5′CTGGGTGTCATGAGCAATGT3′ and ZFN-R 5′AGGAGGCTGAGGAGTGACCT3′ primers, electrophoresed, gel purified using the QIAquick Gel Extraction Kit (Qiagen), and screened for mutations using the Cel1 nucleotide mismatch assay (Sigma). The founders were confirmed by Sanger sequencing (Retrogen). *Tbc1d20*^*ZFN/+*^ did not differ phenotypically from *Tbc1d20*^*+/+*^ mice and both genotypes were used as controls. RNA was isolated from spleen, kidney, liver, and testes and the *Tbc1d20* transcript was reverse transcribed, PCR-amplified and sequenced as previously described [5]. Comparative sequence analysis was performed using DNAStar software. Allelic breedings utilized *bs/+* mice previously obtained from Jackson Laboratories and the *bs* allele was genotyped as previously described [5]. The treatment and use of all animals in this study was compliant with all protocols and provisions approved by the Institutional Animal Care and Use Committee (IACUC) at the Medical College of Wisconsin.

### Clinical evaluations, histology, and immunohistochemistry

Mouse eyes were examined with a Topcon SL-D8Z slit lamp biomicroscope with a Nikon SLR-based Photo Slit Lamp imaging system following mydriasis with 1% Atropine Sulfate (Bausch & Lomb). Eyes, brains, and testes were collected at 8 weeks of age. Eyes and testes were fixed in 4% paraformaldehyde (PFA), paraffin embedded and H&E stained as previously described [5]. Brains were fixed at 4°C for 24 h in 4% PFA followed by 30% sucrose for 24-72 hrs. Brains were then sectioned at 30 μm on a sliding microtome (Leica) and stained with DAPI to label all nuclei. Immunostaining was done with TRA54 (B-Bridge) as a primary antibody and DyLight 488 goat anti-rat (Abcam) as a secondary antibody following the manufacturer’s recommendations. PNA staining was performed utilizing the Lectin PNA-Alexa-488 conjugate (Life Technologies) according to the manufacturer’s recommendations. Slides were DAPI stained according to the manufacturer’s recommendations (Life Technologies), mounted using Fluoromount-G (Southern Biotech), and imaged using a Nikon DS-Fi1 camera on a Nikon Eclipse 80i microscope using NIS-Elements software (Nikon).

### Functional analysis of the *Tbc1d20*^*ZFN*^*allele*

To generate an N-terminal FLAG-tagged *Tbc1d20* clone, *Tbc1d20 (*BC034504.1) clone MGC: 25843/IMAGE: 4192736 (Open Biosystems) was PCR-amplified utilizing PCR primers (F 5′AAGCTTGCGGCCGCGGCCCTCCGGCCCTCAAAG3′ and R 5′GGATCCTCTAGATTAGGGGAACAGCTGCAGCTG3) to incorporate a 5′ NotI restriction site and 3′ XbaI site. The PCR product was subcloned via directional ligation into the *NotI* and *XbaI* sites in the MCS of pFLAG-CMV-2 (Sigma-Aldrich). Mutagenesis to introduce the *ZFN* deletion was performed with the Phusion Site-Directed Mutagenesis Kit (Finnzymes) using F5′Phos-CAGGGCTACCATGACATCGTGGTCACATTT3′ and R5′Phos-GAGCTGAGGGTTGCGATCCAGGACGAGGAG3′ primers. Generated clones were confirmed by Sanger sequencing.

HeLa cells were cultured in DMEM containing 10% fetal bovine serum at 37°C and 5%CO_2_. For transfections, HeLa cells were grown on glass slides in 12-well plates and transfected with Lipofectamine LTX (Life Technologies) following the manufacturer’s recommendations. Following transfections, the coverslips were washed with 1XPBS, then fixed with 4% PFA in PBS pH7.4 for 15 minutes at room temperature, washed with ice cold 1XPBS, permeabilized with 0.25% Triton X-100 in PBS (PBST), and then washed with 1X PBS for 3X5 minutes. The coverslips were immunostained with FLAG (Sigma) and GM 130 (Abcam) antibodies overnight at 4°C and for 1 hr at RT, with Alexa 488 and 546-conjugated (Life Technologies) secondary antibodies following the manufacturer’s recommendations. The coverslips were stained with DAPI for 5 min, washed with 1XPBS, mounted onto glass slides with Fluoromount-G mounting medium, and photographed with a Nikon DS-Fi1 camera on a Nikon Eclipse 80i microscope.

### Mouse embryonic fibroblasts (MEFs)

MEFs were isolated from the E13.5 mouse embryos (from the *Tbc1d20*^*ZFN/+*^*X Tbc1d20*^*ZFN/+*^ cross) that genotyped either *Tbc1d20*^*ZFN/ZFN*^*or Tbc1d20*^*+/+*^ and were maintained as previously described [5,21]. Lipid droplets were evaluated as described previously utilizing media supplemented with 400 μM oleic acid (Sigma Aldrich) for 24 h and stained with 1 μg/μL BODIPY 493/503 (Life Technologies) [5]. All slides were mounted using Vectashield with DAPI (Vector Labs). Imaging was done with a Nikon DS-Fi1 camera on a Nikon Eclipse 80i microscope using NIS-Elements software (Nikon). Quantification of the lipid droplets was performed as previously described [22] using ImageJ (US National Institutes of Health) and NIS-Elements software. For each analysis, at least 20 cells per genotype were evaluated and statistical significance was determined by a *t*-test (Graphpad Prism) where p < 0.05 was treated as significant. For Golgi analysis, the control and *Tbc1d20*^*ZFN/ZFN*^ MEFs were immunostained using GM130 (Abcam) primary antibody and Alexa 488-conjugated secondary antibody (Life Technologies) following manufacturers’ recommendations. Western blots were run using cell lysates generated from control and *Tbc1d20*^*ZFN/ZFN*^ MEFs following lysis with RIPA buffer supplemented with a protease inhibitor cocktail (Sigma). Cell lysates were immunoblotted with GM130 (BD Biosciences) primary antibody and HRP-conjugated secondary antibody (Abcam) following the manufacturer’s recommendations as previously described [5]. Even loading was established following immunoblotting with β-actin HPR conjugated antibody (Abcam). The detection was performed using the ECL Western Blot Analysis System (Amersham) following the manufacturer’s instructions.

## Abbreviations

WARBM4: Warburg Micro syndrome 4
*bs*: blind sterile
ZFN: Zinc finger nuclease
WARBM: Warburg Micro syndrome
GAP: GTPase activating protein
PNA: Peanut agglutinin
LDs: Lipid droplets
